# Options for agricultural service cooperatives in a postsocialist economy: Evidence from Romania

**DOI:** 10.1177/0030727019861973

**Published:** 2019-07-10

**Authors:** Axel Wolz, Judith Möllers, Marius Mihai Micu

**Affiliations:** 1 Department of Agricultural Policy, Leibniz Institute of Agricultural Development in Transition Economies, Halle (Saale), Germany; 2 Faculty of Management, Economic Engineering and Rural Development, University of Agronomic Sciences and Veterinary Medicine of Bucharest, Bucharest, Romania

**Keywords:** Agricultural service cooperatives, private farming, role of government, policy change

## Abstract

Almost three decades since the collapse of the socialist regime, Romania’s farm structure is characterized by a distinct dual pattern. The far majority of farms is relatively small, while a small number manages about half of the total utilized agricultural area. Most farmers face significant constraints in creating viable farm businesses. When this is the case, it can be assumed that farmers will unite and establish agricultural service cooperatives (ASCs), as has been observed in many other parts of the world. In Romania, however, as in many other postsocialist economies, farmers tend to be reluctant to form or join formal organizations of mutual assistance. Yet there are signs of change, as first ASCs have recently been established. The objectives of this contribution are twofold: First, we discuss the major obstacles why ASCs did not develop after regime change. Second, we analyze the major reasons and influencing factors why private family farmers become more open to this type of formal organization in recent years. The analysis is based on a literature review, farm statistics, and qualitative in-depth interviews with farmers in 2018.

## Introduction

In Romania, as in most other postsocialist economies, the newly established private family farmers show a strong psychological resistance to forming or joining formal organizations of mutual assistance. In general, this can be attributed to a considerable lack of trust (Balint and Wobst, 2006; Möllers et al., 2018). However, since a short period first changes in this respect can be observed. In this exploratory analysis, we will discuss the major obstacles to farmers in establishing self-help organizations, such as agricultural service cooperatives (ASCs), and the major influencing factors why this is just changing. Hence, we aim to achieve a better understanding of why cooperation has yet to find success among farmers in postsocialist economies. This is an extremely relevant, but underresearched field. Evidence on the individual motivation, reasons, and actual factors that lead to farmers to form or join organizations of mutual assistance is patchy at best (Bouamra-Mechemache and Zago, 2015; Gijselinckx and Bussels, 2014).

For postsocialist economies, Grashuis and Su (2019) in their recent comparative analysis concluded that agricultural cooperative development is an underresearched topic. To our knowledge, this article represents the first research in Romania about agricultural cooperative development in recent years. It combines several sources to better understand the issue at hand. For a deeper insight into the rationale of farmers, we draw on key informant interviews, which were conducted during summer and autumn 2018 in Bucharest and southwest Romania with individual farmers (members and nonmembers of ASCs), group leaders, and public officials at national and regional levels. A broad outline of open questions was used. In addition, one of the authors who is (part-time) farmer himself is heavily involved in farmers’ associations and cooperative promotion. He is having informal talks and discussions with fellow farmers and government officials about this issue on a continuous basis since quite a number of years. The empirical data are complemented by a literature review and national statistics.

The following section provides background information on the Romanian farm structure and a general discussion about the need for cooperation among private family farmers. Next, we discuss the role of informal collaboration. The major part of this contribution is focusing on the relevant obstacles that hinder that formal cooperation is more popular in Romania, followed by a section focusing on the major reasons why private farmers have recently become more open to the formation of ASCs. A final section concludes.

## Emerging dual farm structure after regime change since the early 1990s

Agriculture plays an important role in the Romanian society and economy; the country once served as the breadbasket of the Ottoman Empire. However, land distribution was highly inequitable. During the 20th century, Romanian agriculture underwent far-reaching changes. After two land reforms following World War I and World War II, respectively, and the collectivization of agriculture during the 1950s, private farming re-emerged during the early 1990s (Sabates-Wheeler, 2005). Similar to the situation before World War I, a dual pattern of farms can once again be observed, that is, a small group of large farms on the one side and a large group of small- and semi-subsistence farms on the other (Table 1). Farms larger than 100 ha account for only 0.4% of all farms but cultivate around 48% of the utilized agricultural area (UAA). Farms cultivating up to 1 ha account for 54% of all farms but manage only around 5.1% of the UAA. The share of medium-sized farms cultivating 10–100 ha is very small (Tudor, 2015; Popovici et al., 2018). While agriculture remains an important sector with respect to its share of national gross domestic product (about 4.3%) and even more so in employment (about 25%), Romania has become a net food importer, particularly with respect to meat, fruits, and vegetables (Agra-Europe, 2017, 2018).

**Table 1.** Farm structure in Romania (2016).

Size group (ha)	Farms		UAA	
	Number	Percentage	Hectare	Percentage
<1	1,850,000	54.1	640,000	5.1
1–10	1,480,000	43.4	4,250,000	34.0
10–100	74,748	2.2	1,630,000	13.1
>100	12,310	0.4	5,970,000	47.8

UAA: utilized agricultural area.

*Source*: National Institute of Statistics (2018). www.insse.ro.

Particularly during the first years of transition, the national economy was on the edge of collapse and the political situation was in permanent turmoil. This led to a highly unpredictable and unstable environment (Calinescu, 2012). The situation also affected agricultural markets, which were characterized by market failures and a high degree of opportunism among buyers, sellers, and regulatory agencies. Many small-scale farms, which were newly established after the regime change, became a social “safety net” against the changes and shocks generated through the process of restructuring the socialist economy. Most smallholder farmers were (and many still are), in fact, excluded from both the input and product markets (Möllers and Bîrhală, 2014) and did not develop into commercial entities. Although many of these farms may not be economically viable, they play an important role in the Romanian society (Mikulcak et al., 2015; Tocco et al., 2014; Tudor, 2015).

## Need for cooperation among private family farmers

Most producers operate in isolation and face multiple constraints that prevent them from taking full advantage of any market opportunity (Cook, 1995; Fischer and Qaim, 2012). There seems to be an urgent need for self-help organizations and, in theory, it could be expected that, faced with such problems, farmers would join forces and collaborate. Evidence from other countries (Bijman, 2016) shows that farmer organizations offer one way for farmers to improve their livelihoods. Organizations can help farmers to overcome the high transaction costs resulting from their relatively small individual sizes; improve access to vital resources, such as inputs, credit, training, and information; and reduce barriers of entry into markets by improving their bargaining power vis-à-vis other market actors (Liverpool-Tasie, 2014; Markelova et al., 2009). Indeed, after the collapse of the socialist regimes, the establishment of cooperative networks seemed to be a natural conclusion at that time. As, for instance, Hristova and Maddock (1993) conclude, “[v]oluntary cooperatives appear to hold considerable attraction for the new land owners” (p. 461).

According to behavioral theory (March and Simon, 1961), individuals voluntarily unite if they believe they can achieve more together than they can individually. Self-determined individuals decide to form or join a group of mutual assistance if the total incentives offered to them by this organization exceed the contributions expected of them. Hence, by intentionally joining a group, individuals expect to be able to utilize the group’s benefits to realize their own needs and interests. The incentives to join cover material as well as immaterial ones. Most empirical studies conclude that the primary motive for membership of self-help organizations is the attainment of economic advantages (Laurinkari, 1994).

The means of cooperation are manifold. Individuals might do so informally in small groups: within families, and among friends and neighbors, or formally, for instance, in the form of ASCs. Formal groups are registered as legal entities. At a certain stage of economic development, formal registration is essential to effectively participating in economic life. One very popular option is to register as ASCs.

Cooperatives can draw on a long history reaching back to the first half of the 19th century. In general, a cooperative is defined as “an autonomous association of persons united voluntarily to meet their common economic, social and cultural needs and aspirations through a jointly owned and democratically controlled enterprise” (ICA, 1995). Individual members are the owners of their cooperatives and, in general, fulfill three major roles: as users/beneficiaries, controllers, and financiers (Golovina and Nilsson, 2011). However, individuals do not only join a business organization to improve their economic well-being, that is, the “cooperative enterprise,” but also become members of a social group, that is, the “cooperative society” (Draheim, 1955). It is an association of individual partners. Hence, social relations among (potential) group members are vital (Fürstenberg, 1994). Individuals will only form or join an organization and stay loyal to it if there is a certain level of interpersonal trust among the members. They must be relatively sure that their comembers will fulfill their obligations and observe their given commitments (Markelova et al., 2009).

While there are many options to differentiate cooperatives (Sykuta and Cook, 2001), following Hagedorn (2014), we just distinguish between two main forms; that is, ASCs on the one side and agricultural production cooperatives (APCs) on the other. The basic difference refers to the fact that in ASCs, private farmers manage their farms independently and patronage the cooperative enterprise, while in APCs farmers are giving up their individual farms and manage their common assets jointly. In principle, both types of cooperatives can be set up on a voluntary basis. However, as Schiller (1969) already observed, APCs are just not attractive for private farmers and not a single one could be identified in any old settled village in Western Europe. Schmitt (1991, 1993) discussed the decollectivization process of collective farms during the early 1990s in postsocialist economies and drawing on transaction cost theory concluded that APCs are not competitive in comparison to corporate farms on the one side, and in particular to family farms on the other. Nevertheless, in some postsocialist economies, collective farms have been transformed into APCs on a voluntary basis, as private farming did not develop as anticipated by reformers (e.g. Wolz et al., 2009, for East Germany). In Romania, no APC can be found these days anymore (Bercu, 2018).

## Informal cooperation among farmers

While in most transition economies, formal organizations of mutual assistance among farmers are lacking and a deficit of trust can be observed, this does not mean that there is no cooperation at all. Many do cooperate informally and help each other in case of need (Abele and Frohberg, 2003; Gardner and Lerman, 2006; Gijselinckx and Bussels, 2014). In Romania, farmers often form small informal groups based on social and familial ties to overcome labor peaks or to exchange information of any kind. Even family members no longer living in rural areas are often included. These members return to the villages over the weekends or during periods when additional help is required on the farm. In general, they are paid in kind, that is, in the form of crops, food, and/or beverages (Sabates-Wheeler, 2005).

A special kind of informal cooperation is joint farming. These informal groups comprise, in general, 4–15 families. Due to the restitution rules, individuals only received access to very small plots and strips of land. Those who want (or need) to earn a certain income from farming cannot cultivate their land effectively at an individual level. With the help of the group, they can achieve higher levels of production. Usually, they unite with close kin. They pool their resources, divide tasks within the group, and specialize in certain activities (crops and livestock) in order to increase their returns to agriculture. Where possible, they unite working members around a relatively highly skilled member (Sabates-Wheeler, 2007). This type of “family association” does not require much from each partner. As informal groups, they do not have to pay any taxes and there is no need to employ staff (Verdery, 2003). Since these informal groups are characterized by their “social lumpiness,” it is difficult to increase their size without encountering problems of labor supervision and moral hazard (Sabates-Wheeler, 2007). While informal groups of mutual assistance are of great relevance, there are no available figures as to their exact numbers or performance.

## Major obstacles to the setup of ASCs in Romania

There seem to be strong theoretic arguments in favor of formal organizations of mutual assistance and farmers do cooperate informally, but still a strong psychological resistance among Romanian farmers as among their colleagues in other postsocialist economies (Gardner and Lerman, 2006) to forming and joining ASCs can be observed. What are the major obstacles hindering farmers just to do so? While there seem to be a number of similarities among the various postsocialist economies, the actual development of formal cooperation at national level is diverse and contrasting (Hagedorn, 2014). Hence, it is almost impossible to generalize these obstacles for all postsocialist economies or even for other regions of the world. In this contribution, we distinguish between historical, structural, mental, and political and institutional obstacles to the establishment of ASCs in Romania.

### Historical obstacles

In Romania, ASCs have a long tradition. Their present image can only be fully understood when this past experience is taken into account. Roughly, three major periods can be distinguished, that is, the pre-socialist, the socialist, and the immediate postsocialist years.

In the Austrian–Hungarian part of the country, the cooperative movement developed in the second half of the 19th century. Farmers mainly of German and Hungarian origin established the first cooperatives. The spirit of ethnic togetherness seems to have been an important element of cooperation (Balint and Wobst, 2006). The former Kingdom of Romania was one of the founding members of the ICA in 1895 (Bercu, 2014). During the interwar period, the state adopted several laws promoting agricultural development. In 1931, the Law on Cooperatives came into effect. However, Cartwright (2001) in his analysis concluded that “agricultural co-operatives…had limited practical impact” (p. 205). They were not organized as self-help organizations, but rather as official institutions controlled, financed, and directed by the state. Hence, few farmers joined them (Cartwright, 2001).

During the socialist period, as was also the case in the other socialist countries, the term “cooperative” was misused to mean the enforced collectivization of family farms. This process began in Romania in May 1949 and, after much resistance from the farmers, was finalized in March 1962 (Iordachi and Dobrincu, 2014). It is argued that the destructive impact of the totalitarian communist legacy persists in all postsocialist economies today (Gijselinckx and Bussels, 2014; Milczarek-Andrzejewska and Śpiewak, 2018), since the collective farms established by coercive means were often labeled as agricultural cooperatives. This experience led to a high degree of distrust among farmers with regard to any type of collective action. In Romania, collective farms “left a bitter taste in the mouth of the rural people” (Verdery, 2003, p. 233). They were regarded as property theft. As such, cooperatives have a very bad image and all types of cooperation where the word “cooperative” is used are seen as a link to the unpopular communist legacy (Calinescu, 2012).

The early postsocialist years were characterized by nonconsistent policies with respect to decollectivization, restructuring of farms, and privatization of agricultural properties. In addition, the overall economic situation deteriorated rapidly. Although the government adopted a law on agricultural cooperatives in 1991 (Law 36/1991), it used the term “agricultural societies” instead of “cooperatives.” In principle, the law provided the legal basis for the decollectivization of collective farms. These societies, however, had nothing in common with the cooperative spirit, which is based on voluntary membership and codetermination rights of members. They simply gave the former collectives a new label. Because managers often performed poorly and lacked ethical conduct, members left whenever possible and their numbers declined rapidly during the1990s and early 2000s (Calinescu, 2012; Tudor, 2015). The law, however, did not provide a legal basis for forming new ASCs.

Due to this negative experience with agricultural cooperatives during the presocialist, socialist, and postsocialist periods, lack of trust seems to be one of the most important reasons that prevented ASCs from being formed after the regime change. Mutual distrust among the inhabitants in many villages, in addition to low levels of human and financial capital, led to the poor exchange of information and skepticism toward new developments (Mikulcak et al., 2015; Sabates-Wheeler, 2005). This attitude has been confirmed by a survey among small-scale farmers in 2013. The level of distrust is very high. Nevertheless, farmers showed interest in joining groups of mutual assistance. However, the lack of trust might be a key constraint when translating intention into actual behavior (Möllers et al., 2018).

### Structural obstacles

In addition, the present farm size structure, as shown in Table 1, might also indicate a structural obstacle. Many studies on cooperation among farmers conclude that not all types of farmers are equally likely to become members. There seems to be a “middle-size bias” when forming cooperatives. The smaller and poorer farmers, as well as the richer and larger farmers, often do not join cooperatives. One explanation might be that this form of collective action is too costly in relation to the potential benefits for the very small farmers, while large farmers can organize their benefits in a more cost-effective manner due to their large size and production volumes (Cagwiza et al., 2016; Fischer and Qaim, 2014; Verhofstadt and Maertens, 2014). This is an important argument when it comes to Romanian situation, as there are not that many farms cultivating 10–100 ha (Tudor, 2015; Popovici et al., 2018).

### Mental obstacles

An alternative school of thought argues that it is the rational choice of the farmers themselves that explains the lack of ASCs. Farmers might highly value their independence and autonomy. Those who seek to cooperate, or need to cooperate, might worry about their reputation, as they are forced to publicly admit their failure to uphold the ideal of an autonomous farmer (Emery, 2015). Similarly, Roger (2014) argued that many farmers in Romania are semisubsistence oriented and have no investment capacity. Their farm products guarantee them direct food supplies and protect them from price fluctuations.

Rather than reverting to the psychological analyses which invoke mistrust inherited from the Communist period, and the view that any form of mutualization is deemed as a first step towards collectivization, this behavior is better explained by a belief in a form of insurance that is at once economic and social. (Roger, 2014, p. 738)

Farmers want to remain independent from others, maintain full control over their assets, and be in a position to face unexpected events. This type of farming might be inefficient in terms of returns to labor and factor inputs, but it assures survival and a basic standard of living (Roger, 2014).

### Political and institutional obstacles

The political and institutional conditions are very influential in setting up and/or running agricultural cooperatives and governments have a vital role. Iliopoulos (2013) identified five main areas of government involvement: (i) the provision of a friendly legal framework that does not discriminate against these formal organizations of mutual assistance; (ii) exemption from antitrust laws and regulations; (iii) beneficial tax treatment when it comes to business activities with members; (iv) access to favorable credit terms; and (v) technical assistance. The degree of involvement varies from country to country but the independence of agricultural cooperatives from government interference in daily management must be strictly observed (Iliopoulos, 2013). During the recent years, agricultural cooperatives seem to have become more attractive to farmers all over the world. In most countries, the governments have not only adopted the legal basis for cooperatives in line with the rules of the ICA but also provided substantial technical and financial support in their establishment. Particularly in situations when farmers were reluctant, did not know how to create them themselves, or were waiting for others to take over the initiative, many governments intervened and promoted the setup of ASCs (Sarker, 2014; Wijen and Ansari, 2007).

In general, it was anticipated that after having given a kick-start, these “top-down” organizations will become genuine self-help organizations over time. The rapid development of ASCs in many Sub-Saharan African countries can be greatly explained by the strong involvement of the individual governments. In many of these cases, financial support is connected with government interference in daily management (e.g. Verhofstadt and Martens, 2014 for Rwanda; Liverpool-Tasie, 2014 for Nigeria; and Bijman et al., 2016 for a general overview with a focus on Sub-Saharan Africa). In these ASCs, the vast majority of the members are the users and beneficiaries of the cooperatives, but they are not the controllers or financers. As Golovina and Nilsson (2011) concluded in their review of government-initiated agricultural cooperatives, in general, these types of top-down initiated organizations are not successful over time. There seem to be no successful management practices available demonstrating how to eventually convert them into businesses controlled and owned by members. On a more macrolevel, when comparing cooperative development among the European Union member states, Brusselaers et al. (2014) concluded that good performing cooperatives currently seem to receive less public support toward maintaining their favorable position.

After the regime change, the Romanian government was slow to provide the proper legal framework for establishing ASCs. As in most other postsocialist economies, ASCs were not on the political agenda. At that time, the principles of liberalism were popular and the political focus was on the protection of individual entrepreneurship and on individual success, while collective action and cooperation had fallen out of fashion. In general, capitalist companies were preferred (Gijselinckx and Bussels, 2014). Indeed, there seems to be a vicious circle. Since there is a small number of ASCs, there is no institutional lobby in favor of them and, hence, there is a lack of political interest in their support. Hence, there is little pressure on governments to design policy measures promoting them (Brusselaers et al., 2014). As Roger (2016) observed, the managers of corporate farms dominate the local and national media, which they are able to use in favor of their own interests, while regularly presenting arguments against smallholders. They have succeeded in imposing their views on agricultural development. An organization that claims to defend a model of agriculture geared toward individual farmers has been absent for a long time.

Around 15 years after regime change, the government finally established a legal framework in accordance with the rules of the ICA (Micu et al., 2016) when it adopted the Law on Agricultural Cooperatives (no. 566) in 2004 and the Law on Cooperatives (no. 1). Membership is open to both private farmers and legal entities. However, farmers were slow in making use of it. The main reason provided for this was that the national tax system was not adapted to the specificities of ASCs. They were taxed as limited liability companies, as there was no differentiation between members’ and nonmembers’ business activities. Cooperatives’ surpluses were taxed as profits and members’ patronage refunds were taxed again (double taxing), a problem which also affects other postsocialist economies, as discussed by Lerman (2013) for Central Asia countries. For potential members, there was no economic advantage to joining a cooperative. Similarly, there were no special support programs for ASCs (Bercu, 2014).

Therefore, the development of ASCs has been very slow. By the end of 2014, the number of operational ASCs stood at 162 (Bercu, 2014). On average, they were quite small. In general, membership was around 14, of which 11 were private farmers and 3 were legal entities. The average annual turnover amounted to around 555,000 RON (or around 125,000 EUR) (Micu et al., 2016). Up to early 2018, the number of registered ASCs was more or less stagnating (Bercu, 2018).

## Recent changes in the development of ASCs in Romania

Just recently, first signs for a cautious change with respect to agricultural cooperative development can be observed. Three notable but seemingly interdependent features have to be acknowledged (Bercu, 2018). During the last years, the government revised the Cooperative Law in making cooperative membership more attractive for farmers. In addition, first umbrella organizations of agricultural cooperatives have been established. Finally, more and more grassroot cooperatives are being registered at local levels. While it is impossible to draw a clear line between cause and effect, it is noteworthy that these developments are happening simultaneously and seem to reinforce each other.

Nevertheless, the basic starting point seems to have been the fact that the government revised the legal conditions to make cooperative membership more attractive. In a first step, a solution to “double taxing” was provided in the form of the revised Cooperative Law (Law 164/2016), which came into effect on July 29, 2016. However, the fiscal code was not adjusted in time. Only with the publication of the circulator by the Finance Office on March 26, 2018, have regulations finally been harmonized. Now, cooperative members do not have to pay any taxes on sales that are marketed via their cooperative during the first 5 years (Bercu, 2018).

Similarly, the first umbrella organizations of ASCs were established. In 2017, the “National Association of Food Cooperatives” (Coop Ro) was founded. It is intended to serve as the national apex organization. For time being, it represents 12 associations from different farm sectors, for example, crops, vegetables, poultry, and fruits. For example, one of its members is the “National Union of Cooperatives in the Crop Sector” (UNCSV), set up in January 2018. This union comprises 13 agricultural cooperatives and 2 affiliated members. It plans to expand membership over time. Coop Ro and UNCSV aim to increase their lobbying activities with respect to the public, parliament, and government. They want to overcome the lack of political interest. But they must begin from scratch. They are still only small and their voice not yet strong, but they have already achieved early successes, including the revision of the Cooperative Law (Bercu, 2018). Coop Ro is a member of the “National Federation PRO AGRO,” which is the umbrella organization of the Romanian agri-food sector, which it represents internationally.

However, the most important changes can be observed at the grassroot level. ASCs seem to become more popular among farmers and new ones are being established. By mid-2018, around 200 ASCs were operational (Bercu, 2018). Two major types of ASCs can be distinguished. On the one side, there are loosely structured ASCs focusing on information exchange. On the other, there are ASCs of a more integrating type which provide additional services to their members like joint input supply and/or joint marketing of agricultural products. An estimated 120 of the active ASCs act as a platform of information exchange. In general, they have the word “cooperative” in their official name. There is a small number of founding members. As such, they retain the decision-making power for themselves. The members pay an annual membership fee. Members do not sign share capital. They are not co-owners of their cooperative but act more as clients. The leaders analyze the markets and bring their members together with potential buyers of agricultural products (i.e. processors, retailers, etc.) as well input providers at special bargaining sessions. The actual deals are conducted between the farmers and the buyers (sellers) on an individual level. In the interviews with farmers, Farmer M stated that he is quite happy with this approach. He is cultivating 220 ha with a focus on crop production and joined this type of cooperative 4 years ago. In his cooperative, there are around 30 members in addition to the 5 founding members. The annual membership fee is 1000 RON (about 250 EUR). With respect to inputs, he estimates that he is saving around 30% compared to if he were acting independently.

This type of loosely structured cooperative providing information exchange might be a first step in setting up a cooperative network over time. The farmers are relatively open to cooperatives and they fully understand that ASCs in these days have nothing in common with the collective farms of the past. However, to establish or join an ASC that jointly manages input provisions and sales of products might be a step too far for many farmers at this stage. Farmer C, who is farming 75 ha, but not a member of a cooperative, does not like the idea of the “compulsory buying of inputs and selling of products.” He is reluctant “to do everything through the cooperative.” He prefers the more casual nature of information provision. In general, farmers are open to the cooperative approach and do not mind using the term “cooperative,” but they are afraid to take control in creating their own cooperatives in their communes. As Farmer J, who is farming 760 ha with his two brothers, puts it:

I totally agree with the cooperative idea. I’d like to have one in this commune. However, I’m afraid to do it on my own as I know it will be a laborious process to convince my fellow farmers about this idea and go through the registration procedure.

Next to the loosely structured model of ASCs, there are around 80 ASCs of a more integrating type. The ASCs are dealing with joined input supply and marketing activities. As farmer MM cultivating 110 ha explains, he and four trusted colleagues established their cooperative in August 2018 as founding members. It aims not only at the bulk marketing of cereals but also at developing new marketing channels for different cereal varieties. The founding members paid 2000 RON as share capital each, while newly joining members just pay a token share capital of 100 RON. To cover running expenses, each member is required to pay an annual membership fee of 2000 RON. Each member is required to market half of the production volume of his/her area cultivated with cereals through the cooperative. Right after setup, this cooperative comprised more than 40 members already. By the end of 2018, the cooperative comprised around 70 members cultivating, on average 100 ha. As Farmer MM clarifies:

It is not our aim to get government subsidies for our own farms, but to establish market power so that we will become an interesting partner for the processing industry. Saving the sales tax during the first five years is definitely another big incentive. But we do not know what will happen after this period.”

He is very optimistic that members will see financial benefits next year already.

In summary, while statistics are scarce, the interview findings show that farmers in Romania are no longer against ASCs. They definitely see the advantages in joining formal groups of mutual assistance. Who are these farmers who pioneer to form ASCs in a setting described by a low level of trust? The interviews show that these farmers have certain characteristics: They are relatively young and well-educated; a large share of them has passed university education. They are very optimistic about the future perspectives of farming, as they see farming as a prospective source of income. Their farms are neither small nor very large-scale; hence, the “middle-size bias” can be observed; they manage farms comprising approximately 100 ha. Nevertheless, they are cautious in transferring the decision-making rights of farm management to a cooperative group. At this stage, most farmers are happy to join more loosely structured ASCs providing information exchange, but supply and marketing cooperatives are cautiously growing as well. They have some trust in fellow farmers, but, in general, do not want to commit themselves too much. However, leadership for setting up cooperatives is very scarce.

In summer 2018, the development of ASCs got a big push. Within less than 2 months, another 400 ASCs were newly registered. Based on our interviews, the major reason for this push seems to be the newly adopted government rule that farmers who are members of a cooperative will receive preferential treatment when applying for investment subsidies for their own individual farms. It is noteworthy that around 90% of these newly registered ASCs are located in only 3 of 41 districts in the country. These districts seem to be very close to the dominant national political party. However, whether these newly established APCs formed primarily because of subsidies will be of a lasting nature is, at least at this stage, doubtful. Nevertheless, we suggest that those ASCs as discussed above form the foundation for cooperative development in Romania.

## Conclusions

Romanian farmers, like most of their counterparts in the other postsocialist economies, used to be reluctant in forming or joining any formal organizations of mutual assistance, like ASCs. While in theory, it can be expected that farmers join forces to improve their well-being, this did not happen. A vicious cycle seemed to be persistent. Farmers lack trust to each other and are not interested in establishing ASCs; therefore, their number was very small. In addition, there were no lobby organizations in pushing for cooperative development. Hence, the government did not feel any need in providing a proper legal framework. In this contribution, we identified historical, structural, mental as well as political and institutional obstacles why ASCs did not develop as it might have been anticipated during the early 1990s.

Only recently, a change in agricultural cooperative development can be observed. There are first signs that this vicious cycle might have been broken. The government provided the necessary legal framework to make cooperative membership attractive for farmers. The number of registered ASCs is increasing since 2018. Two major types of ASCs can be distinguished; the more loosely structured ones focusing on information exchange and the more integrative ones providing additional services. At this stage, most new members of cooperatives seem to prefer the more loosely structured ones. But input supply and marketing cooperatives are cautiously growing as well. When looking at the members who are pioneering cooperative development in an environment of low trust, they share common characteristics. In general, they are relatively young and are well educated; many of them have a university degree. They develop some trust in their fellow farmers. They see a future potential in agriculture and cultivate around 100 ha. In addition, the first umbrella associations of agricultural cooperatives have been established. Leadership at both local and national levels is a scarce factor. Therefore, we conclude that first steps in creating a viable cooperative system have been taken. However, it will take time before cooperatives become strong actors in Romanian agriculture.

## References

[bibr1-0030727019861973] Abele S and Frohberg K (eds.) (2003) Subsistence Agriculture in Central and Eastern Europe: How to Break the Vicious Cycle?, Vol. 22 Halle: IAMO (Studies on the Agricultural and Food Sector in Central and Eastern Europe).

[bibr2-0030727019861973] Agra-Europe (2017) Rumänien: Jeder Vierte arbeitet in der Landwirtschaft *[*Romania: Every Fourth Persons Employed by the Agricultural Sector]. Bonn, Länderberichte: Agra-Europe.

[bibr3-0030727019861973] Agra-Europe (2018) Rumänisches Außenhandelsdefizit bei Agrargütern gestiegen *[*Romanian Trade Deficit Concerning Agrarian and Food Products Increased]. Bonn, Markt und Meinung: Agra-Europe.

[bibr4-0030727019861973] Balint B Wobst P (2006) Institutional factors and market participation by individual farmers: the case of Romania. Post-Communist Economies 18(1):101–121.

[bibr5-0030727019861973] Bercu F (2014) Evolution of agricultural cooperatives in Romania in 2014 Munich, Munich Personal RePEc Archive. MPRA Paper No. 61757. Available at: https://mpra.ub.uni-muenchen.de/61757/ (accessed 13 April 2016).

[bibr6-0030727019861973] Bercu F (2018) President of the National Association of Agricultural Cooperatives in Romania (COOP-RO), personal communication, 14 June 2018.

[bibr7-0030727019861973] Bijman J (2016) The changing nature of farmer collective action In Bijman J Muradian R Schuurman J (eds.) Cooperatives, Economic Democratization and Rural Development. Cheltenham: Edward Elgar, pp. 1–22.

[bibr8-0030727019861973] Bijman J Muradian R Schuurman J (2016) Transformation, inclusiveness and tensions of cooperatives: synthesis and further research In: Bijman J Muradian R Schuurman J. (eds.) Cooperatives, Economic Democratization and Rural Development. Cheltenham: Edward Elgar, pp. 276–288.

[bibr9-0030727019861973] Bouamra-Mechemache Z Zago A (2015) Introduction: collective action in agriculture. European Review of Agricultural Economics 45(5):707–711.

[bibr10-0030727019861973] Brusselaers J Poppe K Azcarate TG (2014) Do policy measures impact the position and performance of farmers’ cooperatives in the EU? Annals of Public and Cooperative Economics 85(4):531–553.

[bibr11-0030727019861973] Cagwiza C Muradian R Ruben R (2016) Cooperative membership and dairy performance among smallholders in Ethiopia. Food Policy 59:165–173.

[bibr12-0030727019861973] Calinescu D (2012) Support for Farmers’ Cooperatives; Country Report Romania. Wageningen: Wageningen UR.

[bibr13-0030727019861973] Cartwright AI (2001) The Return of the Peasant: Land reform in post-Communist Romania. Aldershot: Ashgate.

[bibr14-0030727019861973] Cook ML (1995) The future of US agricultural cooperatives: a neo-institutional approach. American Journal of Agricultural Economics 77(5):1153–1159.

[bibr15-0030727019861973] Draheim G (1955) Die Genossenschaft als Unternehmungstyp *[*The Cooperative Association as a Business Enterprise], 2nd ed Göttingen: Vandenhoeck and Ruprecht.

[bibr16-0030727019861973] Emery SB (2015) Independence and individualism: conflated values in farmer cooperation? Agricultural Human Values 32(1):47–61.

[bibr17-0030727019861973] Fischer E Qaim M (2012) Linking smallholders to markets: determinants and impacts of farmer collective action in Kenya. World Development 40(6):1255–1268.

[bibr18-0030727019861973] Fischer E Qaim M (2014) Smallholder farmers and collective action: What determines the intensity of participation? Journal of Agricultural Economics 65(3):683–702.

[bibr19-0030727019861973] Fürstenberg F (1994) Group, the cooperative In: Dülfer E (ed.) International Handbook of Cooperative Organizations. Göttingen: Vandenhoeck and Ruprecht, pp. 409–412.

[bibr20-0030727019861973] Gardner B Lerman Z (2006) Agricultural cooperative enterprise in the transition from socialist collective farming. Journal of Rural Cooperation 34(1):1–18.

[bibr21-0030727019861973] Gijselinckx C Bussels M (2014) Farmers’cooperatives in Europe: social and historical determinants of cooperative membership in agriculture. Annals of Public and Cooperative Economics 85(4):509–530.

[bibr22-0030727019861973] Golovina S Nilsson J (2011) The Russian top-down organised co-operatives—reasons behind the failure. Post-Communist Economies 23(1):55–67.

[bibr23-0030727019861973] Grashuis G Su Y (2019) A review of the empirical literature on farmer cooperatives: performance, ownership and governance, finance and member attitude. Annals of Public and Cooperative Economics 90(1):77–102.

[bibr24-0030727019861973] Hagedorn K (2014) Post-socialist farmers’ cooperatives in central and Eastern Europe. Annals of Public and Cooperative Economics 85(4):555–577.

[bibr25-0030727019861973] Hristova M Maddock N (1993) Private agriculture in Eastern Europe. Food Policy 18:459–462.

[bibr26-0030727019861973] International Cooperative Alliance (ICA) (1995) Definition of a cooperative. Available at: https://www.ica.coop/en/cooperatives/cooperative-identity (accessed 25 March 2019).

[bibr27-0030727019861973] Iliopoulos C (2013) Public policy support for agricultural cooperatives: an organizational economics approach. Annals of Public and Cooperative Economics 84(3):241–252.

[bibr28-0030727019861973] Iordachi C Dobrincu D (2014) The collectivization of agriculture in Romania, 1949–1962 In: Iordachi C Bauerkämper A (eds.) The Collectivization of Agriculture in Communist Eastern Europe. Budapest: Central European University Press, pp. 251–292.

[bibr29-0030727019861973] Laurinkari J (1994) Motivation for Cooperation In: Dülfer E (ed.) International Handbook of Cooperative Organizations. Göttingen: Vandenhoeck and Ruprecht, pp. 620–622.

[bibr30-0030727019861973] Lerman Z (2013) Cooperative Development in Central Asia. Budapest: FAO Regional Office for Europe and Central Asia (Policy Studies on Rural Transition No. 2013-4).

[bibr31-0030727019861973] Liverpool-Tasie LSO (2014) Farmer groups and input access: when membership is not enough. Food Policy 46(1):37–49.

[bibr32-0030727019861973] March JG Simon HA (1961) Organizations, 3rd ed New York: Wiley.

[bibr33-0030727019861973] Markelova H Meinzen-Dick R Hellin J, et al. (2009) Collective action for smallholder market access. Food Policy 34(1):1–7.

[bibr34-0030727019861973] Micu AR Alecu IN Micu MM (2016) Analysis of Romanian agricultural cooperatives structure in 2014 Munich, Munich Personal RePEc Archive, MPRA Paper No. 69475. Available at: https://mpra.ub.uni-muenchen.de/69475/ (accessed 13 April 2016).

[bibr35-0030727019861973] Mikulcak F Haider JL Abson DJ, et al. (2015) Applying a capitals approach to understand rural development traps: a case study from post-socialist Romania. Land Use Policy 43:248–258.

[bibr36-0030727019861973] Milczarek-Andrzejewska D Śpiewak R (2018) Farmers’ associations: their resources and channels of influence. Evidence from Poland. Sociologia Ruralis 58(4):825–845.

[bibr37-0030727019861973] Möllers J Bîrhală BA-M (2014) Community supported agriculture: a promising pathway for small family farms in Eastern Europe? A case study from Romania. Applied Agricultural and Forestry Research 64(3/4):139–150.

[bibr38-0030727019861973] Möllers J Traikova D Bîrhală BA-M, et al. (2018) Why (not) cooperate? A cognitive model of farmers’ intention to join producer groups in Romania. Post-Communist Economies 30(1):56–77.

[bibr39-0030727019861973] Popovici E-A Mitracă B Mocanu I (2018) Land concentration and land grabbing: implications for the socio-economic development of rural communities in south-eastern Romania. Outlook on Agriculture 47(3):204–213.

[bibr40-0030727019861973] Roger A (2014) “Romanian Peasants” into “European Farmers”? Using statistics to standardize agriculture. Development and Change 45(4):732–752.

[bibr41-0030727019861973] Roger A (2016) Power in the field. Explaining the legitimisation of large-scale farming in Romania. Sociologia Ruralis 56(2):311–328.

[bibr42-0030727019861973] Sabates-Wheeler R (2005) Cooperation in the Romanian Countryside: An Insight into Post-Soviet Agriculture. Lanham: Lexington.

[bibr43-0030727019861973] Sabates-Wheeler R (2007) Safety in small numbers: local strategies for survival and growth in Romania and the Kyrgyz republic. Journal of Development Studies 43(8):1423–1447.

[bibr44-0030727019861973] Sarker A (2014) Federated rural organization for governing the commons in Japan. Journal of Rural Studies 36(1):42–51.

[bibr45-0030727019861973] Schiller OM (1969) Cooperation and Integration in Agricultural Production. London: Asia Publishing House.

[bibr46-0030727019861973] Schmitt G (1991) Why is the agriculture in advanced Western economies still organized by family farms? Will this continue to be so in the future? European Review of Agricultural Economics 18(3–4):443–458.

[bibr47-0030727019861973] Schmitt G (1993) Why collectivization of agriculture in socialist countries has failed: a transaction cost approach In: Kislev Y (eds.) Agricultural Co-operatives in Transition. Boulder: Westview Press, pp. 143–159.

[bibr471-0030727019861973] Sykuta ME Cook ML (2001) A new instrumental economic approach to contracts and cooperatives American Journal of Agricultural Economics 83(5):1273–1279.

[bibr48-0030727019861973] Tocco B Davidova S Bailey A (2014) Labour adjustments in agriculture: evidence from Romania. Studies in Agricultural Economics 116(1):67–73.

[bibr49-0030727019861973] Tudor MM (2015) Small scale agriculture as a resilient system in rural Romania. Studies in Agricultural Economics 117(1):27–34.

[bibr50-0030727019861973] Verdery K (2003) The Vanishing Hectare: Property and Value in Postsocialist Transylvania. Ithaca: Cornell University Press.

[bibr51-0030727019861973] Verhofstadt E Maertens M (2014) Smallholder cooperatives and agricultural performance in Rwanda: do organizational differences matter? Agricultural Economics 45(S1):39–52.

[bibr52-0030727019861973] Wijen F Ansari S (2007) Overcoming inaction through collective institutional entrepreneurship: insights from regime theory. Organizational Studies 28(7):1079–1100.

[bibr53-0030727019861973] Wolz A Kopsidis M Reinsberg K (2009) The transformation of agricultural production cooperatives in east Germany and their future. Journal of Rural Cooperation 37(1):5–19.

